# TVIR: a comprehensive vegetable information resource database for comparative and functional genomic studies

**DOI:** 10.1093/hr/uhac213

**Published:** 2022-09-19

**Authors:** Tong Yu, Xiao Ma, Zhuo Liu, Xuehuan Feng, Zhiyuan Wang, Jun Ren, Rui Cao, Yingchao Zhang, Fulei Nie, Xiaoming Song

**Affiliations:** School of Life Sciences/Library, North China University of Science and Technology, Tangshan, Hebei 063210, China; School of Life Sciences/Library, North China University of Science and Technology, Tangshan, Hebei 063210, China; School of Life Sciences/Library, North China University of Science and Technology, Tangshan, Hebei 063210, China; Food Science and Technology Department, University of Nebraska-Lincoln, Lincoln, NE 68588, USA; School of Life Sciences/Library, North China University of Science and Technology, Tangshan, Hebei 063210, China; Institute of Vegetables and Flowers, Chinese Academy of Agricultural Sciences, Beijing 100081, China; School of Life Sciences/Library, North China University of Science and Technology, Tangshan, Hebei 063210, China; School of Life Sciences/Library, North China University of Science and Technology, Tangshan, Hebei 063210, China; School of Life Sciences/Library, North China University of Science and Technology, Tangshan, Hebei 063210, China; School of Life Sciences/Library, North China University of Science and Technology, Tangshan, Hebei 063210, China; Food Science and Technology Department, University of Nebraska-Lincoln, Lincoln, NE 68588, USA; School of Life Science and Technology, University of Electronic Science and Technology of China, Chengdu 610054, China

## Abstract

Vegetables are an indispensable part of the daily diet of humans. Therefore, it is vital to systematically study the genomic data of vegetables and build a platform for data sharing and analysis. In this study, a comprehensive platform for vegetables with a user-friendly Web interface—The Vegetable Information Resource (TVIR, http://tvir.bio2db.com)—was built based on the genomes of 59 vegetables. TVIR database contains numerous important functional genes, including 5215 auxin genes, 2437 anthocyanin genes, 15 002 flowering genes, 79 830 resistance genes, and 2639 glucosinolate genes of 59 vegetables. In addition, 2597 N6-methyladenosine (m6A) genes were identified, including 513 writers, 1058 erasers, and 1026 readers. A total of 2 101 501 specific clustered regularly interspaced short palindromic repeat (CRISPR) guide sequences and 17 377 miRNAs were detected and deposited in TVIR database. Information on gene synteny, duplication, and orthologs is also provided for 59 vegetable species. TVIR database contains 2 346 850 gene annotations by the Swiss-Prot, TrEMBL, Gene Ontology (GO), Pfam, and Non-redundant (Nr) databases. Synteny, Primer Design, Blast, and JBrowse tools are provided to facilitate users in conducting comparative genomic analyses. This is the first large-scale collection of vegetable genomic data and bioinformatic analysis. All genome and gene sequences, annotations, and bioinformatic results can be easily downloaded from TVIR. Furthermore, transcriptome data of 98 vegetables have been collected and collated, and can be searched by species, tissues, or different growth stages. TVIR is expected to become a key hub for vegetable research globally. The database will be updated with newly assembled vegetable genomes and comparative genomic studies in the future.

## Introduction

Vegetables are necessary for human health as they provide rich sources of dietary fiber, minerals, vitamins, and other nutrients [1–4]. The decreased cost of sequencing has led to numerous vegetable genomes being released in the past decade. In particular, the amount and quality of vegetable genomes have markedly increased due to the emergence of third-generation sequencing in recent years [5]. The first vegetable genome to be sequenced was that of *Cucumis sativus* (cucumber) in 2009, and since then the genomes of several vegetable species have been sequenced [6]. For example, two high-quality and chromosome-level genomes of celery and coriander were assembled in our laboratory [7, 8]. Genome sizes vary significantly among different vegetable species. The assembled genome size of garlic (*Allium sativum*) was 16.24 Gb, which was similar that of onion (*Allium cepa*) (14.90 Gb) [9, 10]. However, the genome size of *Neoporphyra haitanensis* (Laver), a lower plant from the phylum Rhodophyta, was only 49.67 Mb. Moreover, pan-genome studies of several vegetable species have been reported recently, including *Brassica rapa*, *Brassica oleracea*, *Brassica napus*, *Raphanus sativus*, *Solanum lycopersicum*, and *Solanum melongena* [11–18].

The availability of these vegetable genomes provides valuable resources for comparative and functional genomics studies, such as the evolution, domestication, and molecular mechanisms of vegetables. The genome sequences of most major vegetable crops have been deposited in public databases, such as the National Center for Biotechnology Information (NCBI) database [19]. However, most genome sequences have not been updated and often lack gene sets and annotation. Currently, several organism-specific databases of vegetables have been constructed, such as the Onion Genome Sequencing Project (https://www.oniongenome.wur.nl) [10], Celery Genome Database (CGD) (http://celerydb.bio2db.com) [7], Coriander Genome Database (http://cgdb.bio2db.com) [8, 20, 21], Radish Genome Database (RadishGD) (http://radish-genome.org/) [22], Pepper Genome Platform (PGP) (http://peppergenome.snu.ac.kr), Eggplant Genome Database (http://www.eggplant-hq.cn/Eggplant/home/index) [23], Lettuce Genome Resource (https://lgr.genomecenter.ucdavis.edu) [24], *Beta vulgaris* Resource (https://bvseq.boku.ac.at/index.shtml) [25, 26], Melonomics (http://melonomics.cragenomica.es) [27], Asparagus Genome Project (http://asparagus.uga.edu/tripal/) [28], Sweet Potato Genome database (http://public-genomes-ngs.molgen.mpg.de/sweetpotato/) [29], White Lupin Genome database (https://www.whitelupin.fr/download.html) [30], and the pan-genome information resource for *B. napus* (BnPIR) (http://cbi.hzau.edu.cn/bnapus/) [31]. In addition, some databases have been constructed for species at the genus or family level, such as the *Brassica* Database (BRAD) (http://brassicadb.cn) [32], The Brassicaceae Genome Resource (TBGR) (http://www.tbgr.org.cn) [33], Solanaceae Genomics Network (SGN) (https://solgenomics.net/) [34], and CuGenDB for Cucurbitaceae (http://cucurbitgenomics.org) [35].

Despite these individual resources, an integrated, comprehensive, and up-to-date database of gene resources for the vegetable community is lacking. Therefore, to make all the vegetable genome sequences, annotated data, and transcriptome information accessible to the vegetable research community, a comprehensive gene resource platform for vegetables— The Vegetable Information Resource (TVIR)—was built in the current study. The purpose of the TVIR platform is to provide a repository that can be used for comparative and functional genomics studies of vegetable species at the whole-genome level. An overview of the TVIR database interfaces, including Browse, Search, Charts, Tools, Download, and Resources, is provided in this report. The database will promote vegetable research by providing several convenient tools and rich omics data resources.

## Results

### Overview of vegetable research

**Figure 1:**
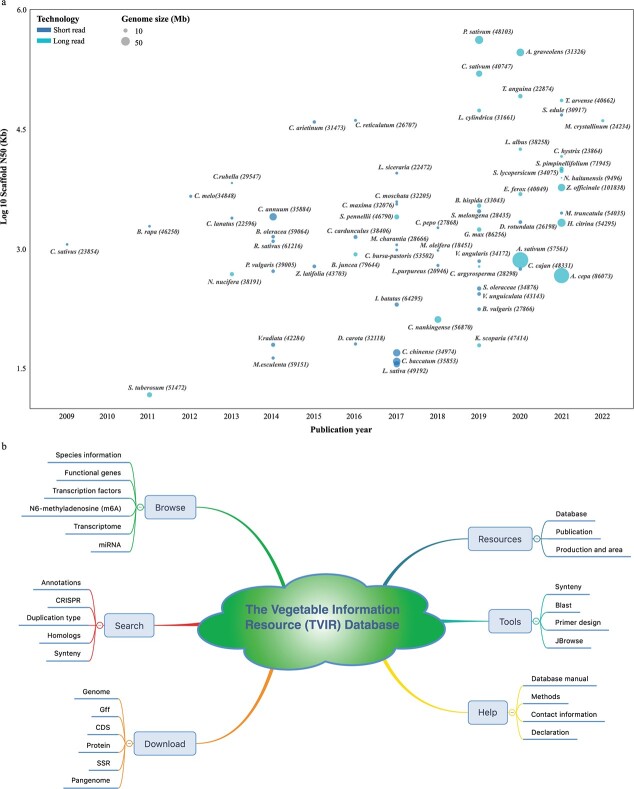
Overview of vegetable genome sequencing and TVIR database. **a** Species information and major genome sequencing indicators (publication year, assembled genome size, gene number, scaffold N50, and sequencing technology) of 65 vegetables from the years 2009 to 2022. **b** Architecture of TVIR database.

According to statistics, the genomes of 65 vegetable species have been sequenced to date (Fig. 1a, Supplementary Data Table S1). In terms of taxonomic distribution, these species comprise 57 eudicots, 6 monocots, 1 basal angiosperm, and 1 Rhodophyta (Supplementary Data Fig. S1a). Among the eudicots, the highest number of species was in the order Cucurbitales (14), followed by Fabales (12), Brassicales (9), and Solanales (9), suggesting their importance to humans. The first vegetable crop to be fully sequenced and assembled was cucumber, released in 2009. Most other vegetable crops have been sequenced in the past 5 years (2017–21), accounting for 67.69% (44) of all assembled vegetable genomes (Fig. 1a, Supplementary Data Fig. S1b).

**Figure 2:**
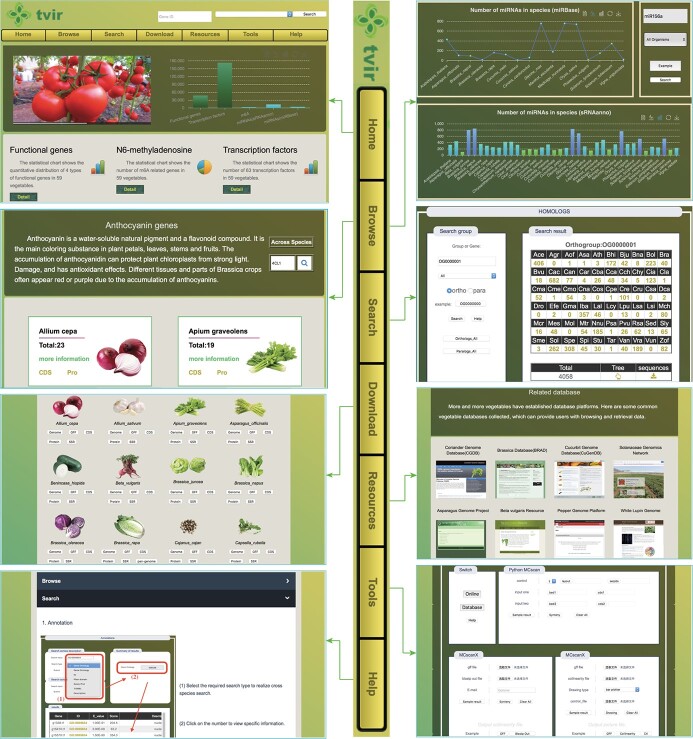
Overview of TVIR database with main interfaces and internal features, including Home, Browse, Resources, Search, Tools, Download, and Help interfaces.

**Figure 3:**
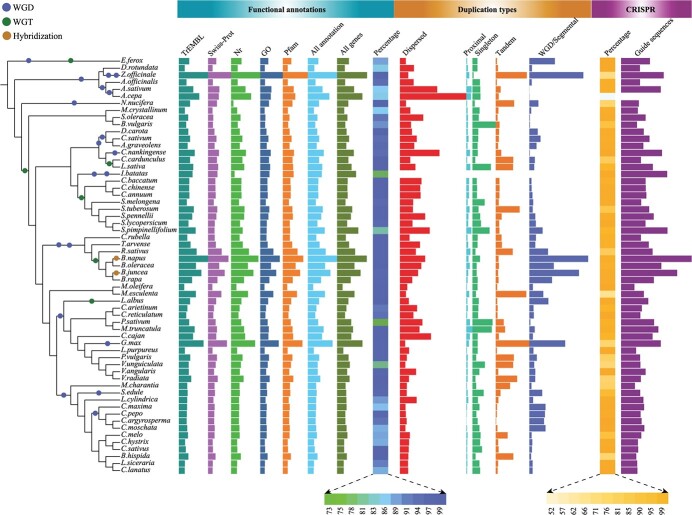
Bar plots of the number of gene functional annotations, gene duplication types, and CRISPR guide sequences in 59 vegetable species. WGD, WGT, and genome hybridization events are indicated by blue, green, and orange circles, respectively. The specific values can be obtained from Supplementary Data Tables S2, S3, and S6.

Related information resources for public genomes from 65 vegetable crops were collected (Supplementary Data Table S1). Due to incomplete genome or gene annotation data for six species, only the remaining 59 species were selected for subsequent analysis.

**Figure 4:**
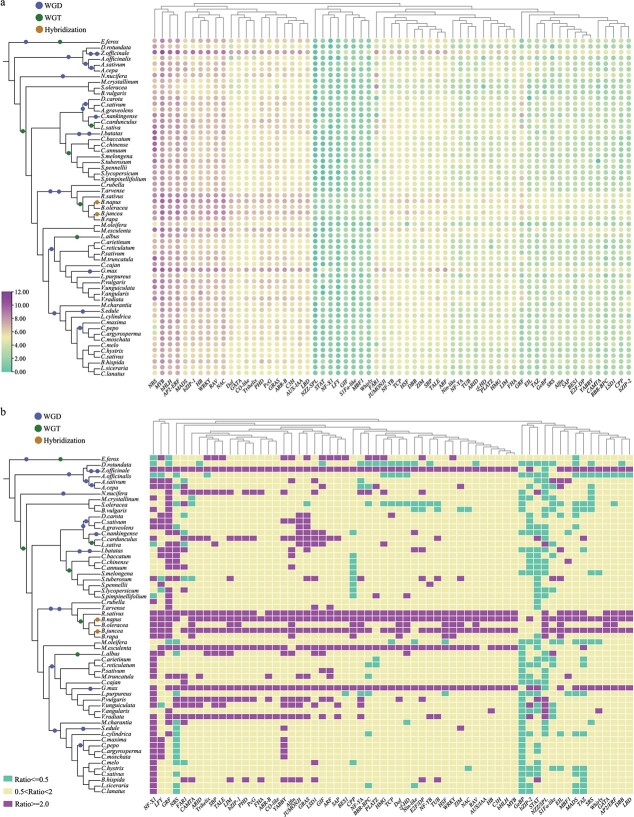
Heat map of TF family analysis in 59 vegetable crops. **a** Circle plot showing the number of members of each TF family in each species. The number of each TF was transformed by log_2_. **b** The ratio of number of members of each TF family compared with *A. thaliana* in each vegetable species. Purple indicates a ratio of ≥ 2, green indicates a ratio of ≤ 0.5, and yellow indicates 0.5 < ratio < 2. The specific values of ratios can be obtained from Supplementary Data Table S8.

### Architecture of TVIR database

Systematic analyses were performed on these collected genomic data, such as gene annotation, orthologous genes, transcription factors (TFs), CRISPR guide sequences, m6A, miRNA, synteny, duplication type, and detection of the main functional genes. Several important functional genes were identified for inclusion in TVIR, including auxin, anthocyanin, flowering, resistance, and glucosinolate genes. Finally, TVIR database was constructed to facilitate access to and employment of these genomic resources and bioinformatic analysis results (Fig. 1b). All the genomic resources were stored in the back-end tables of MySQL, which could be easily accessed through the front-end web application. A detailed description of TVIR database, including Search, Browse, Tools, Download, Resources, and Help interfaces (Figs 1b and 2) is provided below.

#### Search

In this section, users can search the gene annotation, CRISPR guide sequences, duplication type, homologs, and synteny for 59 vegetable species. Based on the five protein databases (Gene Ontology, Nr, Pfam, Swiss-Prot, and TrEMBL), 73.02% (*Pisum sativum*) to 99.88% (*Benincasa hispida*) of all genes were annotated in each species (Fig. 3, Supplementary Data Table S2). All annotations can be searched according to the gene identifier in TVIR.

**Figure 5:**
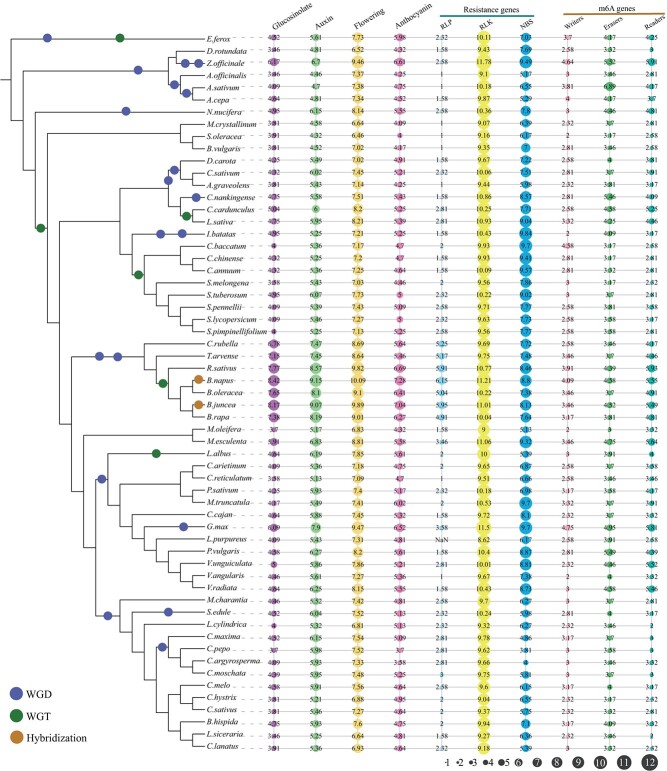
Plot of the number of members of several functional gene families, including auxin, anthocyanin, flowering, glucosinolate, resistance, and m6A genes, in the 59 vegetable crops. The numbers for each gene family were transformed by log_2_. Circle size represents the size of the value, and specific values can be obtained from Supplementary Data Table S9.

To facilitate gene-editing research on vegetable species, CRISPR guide sequences for all genes were designed and deposited in TVIR. In total, 2 101 501 specific CRISPR guide sequences were designed in all examined species, and the success rate of guide sequence design in all species ranged from 52.95% (*Manihot esculenta*) to 99.73% (*Cucurbita argyrosperma*) (Fig. 3, Supplementary Data Table S3).

To explore gene evolution in each vegetable species, homologous gene analysis was performed using the OrthoFinder program. There were 78 256 orthogroups detected among the 59 vegetable species, of which 30 388 groups were species-specific (Supplementary Data Tables S4 and S5). In addition to displaying these orthogroups and downloading sequences from TIVR database, phylogenetic trees reconstructed using the genes in each group were also illustrated. The species tree was reconstructed using orthologous genes to uncover the phylogenetic relationships of these vegetable species.

To clarify the situation of gene duplication or loss after whole-genome duplication (WGD) or whole-genome triplication (WGT) events in vegetable species, syntenic analyses were conducted using amino acid sequences within species or between any two species. The syntenic genes and corresponding figures were then integrated into TVIR database. Furthermore, five duplication types, including singleton, proximal, tandem, dispersed, and WGD/segmental, were detected for each gene in the 59 vegetable species (Fig. 3, Supplementary Data Table S6), and WGD/segmental duplication was dominant in several species that underwent WGD or WGT events, especially for recent genome duplication events. For example, WGD/segmental repeat accounted for the highest proportion of duplication type in *Euryale ferox* (66.57%), followed by *Brassica juncea* (65.32%) and *B. napus* (60.99%) (Fig. 3, Supplementary Data Table S6). This is because *E. ferox* has experienced ancient WGD and recent WGT events [36]. *B. juncea* and *B. napus* not only experienced ancient WGD and recent WGT events, but also tetraploids were formed by diploid hybridization [37, 38]. The proportion of WGD/segmental repeat type in four Cucurbitaceae species (*C. argyrosperma*, *Cucurbita maxima*, *Cucurbita moschata*, and *Cucurbita pepo*) was also >50% due to their recent WGD event [39–41]. Similarly, the proportion of WGD/segmental repeat type was >50% in *B. rapa*, *Lupinus albus*, and *Zingiber officinale* because they underwent a recent WGT event or two rounds of recent WGD events [30, 42–44].

#### Browse

The Browse section provides species information and sequences of functional genes, TFs, m6A, transcriptome, and miRNAs of related vegetable species. For user convenience, the information in the Browse interface is presented according to the family and species. Comprehensive information for each species is provided, including taxonomy ID, common name, classification, chromosome number, genome size, and species pictures. These resources can help users rapidly understand the related vegetable species.

TFs have critical roles in various stresses as well as plant growth and development [45, 46]. In total, 172 493 TFs from 63 families were identified in all examined species (Fig. 4a, Supplementary Data Table S7). The four TF families with the largest number of genes were myeloblastosis (MYB) (18797), nucleotide binding sites (NBSs) (15053), APETALA2/ethylene-responsive element binding factors (AP2/ERF) (11992), and basic helix–loop–helix (bHLH) (10609). Compared with *Arabidopsis thaliana*, the NF-X1, LEAFY (LFY), and growth-regulating factor (GRF) gene families were significantly expanded, while the glabrous-enhancer-binding protein (GeBP), basic leucine zipper 2 (bZIP-2), signal transducer and activator of transcription (STAT), and NOZZLE/SPOROCYTELESS (NZZ/SPL) gene families were contracted in most examined species (Fig. 4b, Supplementary Data Table S8). Almost all TF families were expanded in *Z. officinale* and *Glycine max*, which underwent one additional recent WGD event and two WGD events, respectively (Fig. 4b) [43, 44, 47].

Notably, one common WGT event occurred in *Brassica* and *R. sativus*, and most TF families were significantly expanded in *R. sativus* but not in *B. rapa* and *B. oleracea* [42, 48, 49]. This might be due to most genes being lost after WGT events, consistent with previous reports [42, 49]. In the two tetraploid species of the genus *Brassica* (*B. napus* and *B. juncea*), most TF families were expanded because they were formed by the crossing of two diploid species of the genus *Brassica* [37, 38] (Fig. 4b).

Several important agronomic-related functional gene families were identified, including 5215 auxin genes, 2437 anthocyanin genes, 15 002 flowering genes, 79 830 resistance genes [563 receptor-like proteins (RLPs), 64 214 receptor-like kinases (RLKs), and 15 053 NBSs], edna and 2639 glucosinolate genes in the genomes of 59 vegetable species (Fig. 5, Supplementary Data Table S9). All of these functional genes play essential roles in the breeding of vegetable species and research on them. Users can quickly search and download related genes directly from TIVR database. For example, all 4-coumarate:CoA ligase 1 (*4CL1*) genes in all species can be searched at the anthocyanin gene browsing interface. Furthermore, the *4CL1* gene list is provided and queried sequences are available for users to download at the bottom of the page. Users can employ these datasets to conduct further comparative analysis among cross-species.

One of the most important RNA modifications in plants is m6A methylation. The function and characterization of m6A are a major focus in current plant research. The genes involved in m6A modification are highly conserved across different plants [50]. Therefore, the m6A genes in vegetable species were identified and deposited in TVIR database. Moreover, the m6A genes were classified into writers, erasers, and readers according to their functions. A total of 513 writers, 1058 erasers, and 1026 readers were identified in the 59 vegetable species (Fig. 5, Supplementary Data Table S9). The writers were further divided into 262 methyltransferase-A with 70-kDa subunit (MTA70), 105 Hakai, 111 Wilms’ tumor 1-associated protein (Wtap), and 35 Virilizer genes. These m6A gene resources in TVIR database will provide valuable guidance for the genetic improvement of vegetable crops by epitranscriptome manipulation.

To facilitate the study of vegetable miRNAs, information on miRNAs was collected from the sRNAanno and miRBase databases (Supplementary Data Table S10). In the sRNAanno database 13 454 miRNAs were identified from 37 vegetables and *A. thaliana*, while in the miRBase database 3913 miRNAs were identified from 15 vegetables and *A. thaliana*. In TVIR database the bar or line charts clearly display the number of miRNAs in each species, making it easier for users to perform comparisons among different vegetable species. Furthermore, the hairpin sequences, mature sequences, and target genes of miRNAs were predicted and can be downloaded from TVIR database. Moreover, the structure of each miRNA is illustrated and shown in TVIR. Publications related to each miRNA and links to the NCBI database are also provided. The multi-select dropdown menu allows users to select species to search miRNAs according to their needs. Users can also select all organisms from the dropdown menu, which will then show the search for a certain miRNA in all species.

In addition to vegetable genome data, the transcriptome data of 98 vegetables were collected and collated. Users can easily and quickly select the corresponding transcriptome data according to the Latin name of the species, the plant tissue, or the settings of different growth and development periods. In the retrieved information table, users are provided with accession numbers and links to transcriptome data, sample information, sequencing library, and author information. Moreover, the informationtable can be downloaded, providing useful data resources for theretrieval, collection, and analysis of gene expression of vegetable species.

#### Tools

Four popular tools—Synteny, Blast, Primer Design, and JBrowse—are included in TVIR database to help users conduct comparative and functional genomic analyses. Two syntenic tools, the Multiple Collinearity Scan toolkit (MCScanX) and Python MCscan, are provided and users can conduct syntenic analyses among species with these tools. Both Python MCscan and MCScanX have online and database modes in TVIR database.

For Python MCscan, users can upload bed and coding sequence (CDS) files for syntenic analysis in the online mode. The configuration files ‘layout’ and ‘seqids’ can be created according to the manual of the Python MCscan program. Depending on the user’s needs, this tool can perform a collinear analysis of two to four species at a time. For database mode, users only need to select two to four species in TVIR database, then the syntenic diagram is rapidly illustrated.

For MCScanX, users can upload Blast and general feature format (gff) files for syntenic analysis in the online mode. The database also provides a visualization using the results of syntenic analysis, including gff and collinear files. Four syntenic types, including dot plotter, circle plotter, bar plotter, and dual synteny plotter, can be obtained by selecting different configuration files. For the database mode, users only need to select two species among the 59 vegetable species, and the corresponding syntenic diagram is rapidly displayed according to the selected drawing type.

The Blast tool was included in TVIR database to facilitate sequence alignment. A user-friendly interface was created and Blast databases were constructed using CDS and protein sequences of the 59 vegetable species. All users can easily perform sequence alignment by uploading a Fasta format file or directly copying sequences to the frame. A Primer Design tool was deposited in TVIR to help researchers design primers for genes in the 59 vegetable species by entering the related gene accession number. Moreover, users can also design primers for their gene sequences with Fasta format. In addition, a JBrowse tool was built to show the genomic sequences and features of vegetable genes. This tool allows users to check the detailed information for selected genes on the corresponding chromosome or scaffold sequences.

#### Download

The genome sequences, gff, CDS, and protein sequences of each vegetable species can be obtained from TVIR database. Moreover, the pan-genomes of seven species, including *B. rapa*, *C. sativus*, *L. albus*, *R. sativus*, *S. lycopersicum*, *S. melongena*, and *Solanum tuberosum*, were also provided. Finally, the simple sequence repeat (SSR) markers were identified from all the coding genes of each species, and they can also be downloaded from TVIR database. All these genomic datasets and resources facilitate rapid and easy comparative genomic analyses of vegetable crops.

#### Resources and Help

The Resources section of TVIR provides information on the database, publication, yield, and planting area of related vegetables. Through the Resources page, users can quickly understand the research value and status of major vegetables, and rapidly obtain relevant vegetable data resources. In the Help section, a detailed manual for each interface of the TVIR database is supplied. Furthermore, e-mail addresses and phone numbers are provided to enable users to easily contact us.

## Discussion

In this study, 65 vegetable species with sequenced genomes were collected, and comparative and functional genomics studies were subsequently performed on the 59 species that had well-annotated genomes. As increasing numbers of vegetable genomes have been sequenced, several species-specific vegetable databases have been built, such as RadishGD for radish [22], CGD for celery [7], CGDB for coriander [8, 20, 21], and EGD for eggplant [23]. Moreover, several databases were constructed for species in the same genera or family, such as BRAD for *Brassica* [32], TBGR for Brassicaceae [33], SGN for Solanaceae (https://solgenomics.net/) [34], and CuGenDB for Cucurbitaceae (http://cucurbitgenomics.org) [35].

Although the above databases provide rich resources for vegetable research, they only contain one or several closely related species. Compared with these databases, TVIR integrates most resources of these websites and provides systematic analysis results using 59 vegetable genomes; consequently, TVIR has a number of advantages over existing databases. Firstly, TVIR database contains comprehensive genomic resources from public genomes of 59 vegetable species, making it the first large-scale collection of vegetable genomic data. Secondly, TVIR contains lots of important functional genes (flowering, resistance, anthocyanin, glucosinolate, and auxin genes), m6A, guide sequences of CRISPR, synteny, and orthologs, which were detected by using a series of bioinformatics analyses. Thirdly, TVIR provides the gene annotation information on the 59 vegetable species from five annotation databases. Finally, TVIR includes the Blast, Primer Design, Synteny, and JBrowse tools, which help users easily conduct comparative genomic analyses in vegetable species. TVIR aims to provide researchers with related information for a queried gene, including sequences, domains, annotation, homologs, synteny, and gene family. This ‘data consumer’-oriented function could help people studying vegetables to obtain an overview of queried genes, and this will be particularly useful when designing experiments to verify gene function. Therefore, several user-oriented software and web servers are provided in TVIR to facilitate gene analysis for vegetable molecular biologists.

In conclusion, TVIR will facilitate both comparative and functional genomic studies in vegetables, and potentially plant species. Researchers can easily retrieve and download the target functional genes for cross-species comparative study. Researchers will be encouraged to submit their genome and transcriptome sequences to TVIR, and technical assistance will be assigned for uploading and handling their sequences. TVIR database will be continuously improved and updated with new omics sequences and comparative genomic tools.

## Materials and methods

### Retrieval of genome and gene annotation resources

Genome sequences, gff files, CDS, and protein sequences of each vegetable species were collected from several major public or private databases. For example, most genome datasets were downloaded from NCBI (https://www.ncbi.nlm.nih.gov), JGI (https://phytozome-next.jgi.doe.gov), CuGenDB (http://cucurbitgenomics.org/), BRAD (http://brassicadb.cn) [32], TBGR (http://www.tbgr.org.cn) [33], the Solanaceae Genomics Network (https://solgenomics.net/), and other related databases for single species (Supplementary Data Table S1). Spliced genes were deleted to avoid redundant sequences using a custom Perl script. A total of 65 vegetable species was retrieved for genome sequencing. Genomic information for these species was collated, including their classification, genome size, gene number, scaffold N50, chromosome number, publication status, sequencing information, and genome access databases (Supplementary Data Table S1).

### Functional annotation of genes

Gene annotations of 59 vegetable species were conducted using five protein databases, including Swiss-Prot and TrEMBL of the UniProt knowledgebase (https://www.uniprot.org) [51], Pfam (v34.0) (http://pfam.xfam.org) [52], Gene Ontology (GO, http://geneontology.org) [53], and the non-redundant protein sequence database (Nr, https://www.ncbi.nlm.nih.gov). All gene annotations are displayed in TVIR database. SSR markers were developed according to our previous reports [54–56].

### Identification of orthologous, paralogous, and xenologous genes

OrthoFinder (v2.0) was used to identify orthologs, paralogs, and xenologs [57]. First, the Blastp program was used to obtain the similarity relationships among the protein sequences in different species (E-value <1e−5). Cluster analysis was then performed using the MCL algorithm (inflation value >1.5), and gene and species trees were built using each gene family of all species.

### Detection of collinearity and duplication types

Collinearity analysis was performed by the MCScanX software with default parameters [58]. First, Blastp was used to search for potential homologous genes (E-value <10^−5^) among different species. Then, the collinearity was detected according to the gff files and Blast results. Finally, collinear relationships were illustrated using TBtools [59]. The duplicate_gene_classifier program from MCScanX was used to predict the duplication types [58].

### Identification of transcription factors and functional genes

The 63 TF gene families were detected, using the Pfam database, from the protein sequences of 59 vegetable species (E-value <1e−5) [52]. The 295 flowering genes of *Arabidopsis* were collected from previous studies and the FLOR-ID database [60–62]. The 73 glucosinolate genes of *Arabidopsi*s were collected from previous reports [49, 63, 64], and the 41 anthocyanin genes and 151 auxin genes of *Arabidopsis* were obtained from the BRAD website [32]. Homologous flowering, glucosinolate, anthocyanin, and auxin genes in other vegetable species were identified by Blastp (E-value <1e−5; identity >60%; score >150) with manual checking. Furthermore, the identified candidate genes were further verified by their domains, and related genes with domains were annotated in TVIR database.

### Detection of resistance genes

Three kinds of resistance (R) genes were predominantly identified NBS, RLK, and RLP genes [65, 66]. The NBS genes of each species were detected by the accession number PF00931 in the Pfam annotation with an E-value <1e−5. RLK genes were obtained from the Pfam annotation by the keyword ‘kinase’; in total, 15 gene families were assigned to the RLK genes. The 56 RLP genes of *Arabidopsis* were downloaded from the BRAD website [32], and homologous genes in other vegetable species were detected by the Blastp program (E-value <1e−5; identity >60%; score >150).

### m6A identification

The m6A genes comprised three groups, including writers, readers [IYT521-B homology (YTH)], and erasers [Alkylation repair protein-B (AlkB)] [50]. Writers had four gene families, including MTA70, WTAP, HAKAI, and VIRILIZER, which were identified by the accession numbers PF05063, PF17098, PF18408, and PF15912, respectively. The YTH genes of readers were detected by the accession number PF04146, and AlkB genes of erasers were detected by the accession number PF13532 of the Pfam annotation.

### miRNA collection and target gene identification

The mature, hairpin sequences, and gff files of miRNAs were downloaded from sRNAanno and miRBase (Release 22.1) [67, 68]. The structure of each miRNA was created using the ViennaRNA package (v2.5.0) with slight modifications in batch [69]. The target genes of each miRNA were predicted using the TargetFinder program [70]. All the above datasets were sorted by in-house Perl scripts so they could be displayed in TVIR.

### Cas9 target sequence design for CRISPR

The CasFinder pipeline was used to design the Cas9 target sites for CRISPR [62]. Firstly, the repetitive genome sequences were screened using the RepeatMasker program for each species [71]. The index was then created for each genome by the Bowtie program [72]. Finally, the scripts CasValue_v2.pl and CasFinder.pl from the CasFinder pipeline were adopted to design the guide sequences for the CRISPR study [62]. The candidate sequence was filtered by in-house Perl scripts to obtain the specific sequence for each gene.

### Database construction

Based on the Django framework, the TVIR database was built with MySQL database management according to our previous reports [33, 73] and using several programming languages, such as Python, JavaScript, HyperText Markup Language (HTML), and Cascading Style Sheets (CSS). Several interactive Web interfaces were constructed to help users conveniently search TVIR and obtain the required information. Echarts was used to show the charts, and is an open-source visualization tool integrated into JavaScript. Vegetable genome and gene sequences were processed by Python or Perl scripts, and some bioinformatics tools were employed to perform comparative genomic analyses.

## Acknowledgements

This work was supported by the Natural Science Foundation for Distinguished Young Scholar of Hebei Province (C2022209010), the National Natural Science Foundation of China (32172583, 31801856), the Natural Science Foundation of Hebei (C2021209005), the Educational and teaching reform research and practice project of North China University of Science and Technology (L21106), and the China Postdoctoral Science Foundation (2020M673188, 2021T140097).

## Author contributions

X.S. conceived the project and was responsible for project initiation. X.S. and T.Y. supervised and managed the project and research. The data collection and bioinformatics analyses were led by X.S., T.Y., X.M., Z.L., Z.W., and X.F. The database construction was led by X.S., T.Y., Z.L., and F.N. The manuscript was organized, written, and revised by X.S., T.Y., X.F., X.M., R.C., Y.Z., and J.R. All authors read and approved the manuscript.

## Data availability

All materials and related data in this study are provided in the TVIR database and supplementary files. Other datasets are available upon request to the corresponding author.

## Conflict of interest

The authors declare no competing interests.

## Supplementary Material

Web_Material_uhac213
