# Supplementary material for: TVIR: a comprehensive vegetable information resource database for comparative and functional genomic studies
Source: Hortic Res. 2022 Sep 19;9:uhac213. doi: 10.1093/hr/uhac213 (PMC9719039; doi:10.1093/hr/uhac213)
Supplement: Web_Material_uhac213 [file web_material_uhac213.zip › 2022-09-01,Supplementary Figure 1.docx]

**Supplementary Figure 1**

**Fig. S1 Overview of the 65 vegetable crops with released genomes.** (a) Classification of 65 species. (b) The number of sequenced genomes each year from 2009 to 2022.
